# One Clinical Picture, Two Clinical Entities: A Case Report and Literature Review of Neurofibromatosis and Hemochromatosis

**DOI:** 10.3390/children13091266

**Published:** 2026-09-18

**Authors:** Lorena Elena Melit, Reka Borka Balas, Florin Tripon, Radu Alexandru Prisca, Tamas Toth, Alexandra Stangaciu, Karina Najjar

**Affiliations:** 1 Department of Pediatrics II, George Emil Palade University of Medicine, Pharmacy, Science and Technology of Targu-Mures, 540142 Targu Mures, Romania; lorena.melit@umfst.ro (L.E.M.); najjar.karina-maria.25@stud.umfst.ro (K.N.); 2 Pediatrics Clinic II, Clinical County Mures Hospital, 540142 Targu-Mures, Romania; alexadra.stangaciu986@gmail.com; 3 Department of Pediatrics I, George Emil Palade University of Medicine, Pharmacy, Science and Technology of Targu-Mures, 540142 Targu Mures, Romania; 4 Pediatrics Clinic I, County Emergency Clinical Hospital of Târgu Mureș, 540136 Targu-Mures, Romania; 5 Department of Genetics and Cellular and Molecular Biology, George Emil Palade University of Medicine, Pharmacy, Science and Technology of Targu-Mures, 540142 Targu Mures, Romania; florin.tripon@umfst.ro; 6 Pediatric Surgery and Orthopedics Clinic, County Emergency Clinical Hospital of Târgu Mureș, 540136 Targu-Mures, Romania; radualexandruprisca@gmail.com (R.A.P.); doctothtamas@gmail.com (T.T.)

**Keywords:** neurofibromatosis, Hemochromatosis, children, genetic testing

## Abstract

**Highlights:**

**What are the main findings?**

• NF1 and HC represent two different clinical entities, but with a similar clinical picture.
• Although rare, the coexistence of NF1 and HC is not impossible, and a patient-centered diagnostic and management approach should be provided in each clinical situation.

**What are the implications of the main findings?**

• All patients with suspected genetic disorders should benefit from a thorough multidisciplinary approach.
• WES should be taken into consideration in patients with contradictory clinical or paraclinical findings.

**Abstract:**

Background: Type I neurofibromatosis (NF1) represents an autosomal dominant inherited genodermatosis predisposing to tumor occurrence, caused by mutations in the *NF1* gene, clinically characterized by the impairment of skin pigmentation, dermal neurofibromas, neuro-psychiatric involvement, and Lisch nodules. The initially defined diagnostic criteria were revised in 2021 to facilitate the diagnosis in young children who present only with skin pigmentation anomalies or a positive family history of NF1. Hemochromatosis (HC) is a genetic disorder consisting of systemic iron overload due to a defect in hepcidin. The mutations are commonly located in the *HFE* gene. The clinical picture is associated with joint pain, hyperpigmentation, hepatomegaly, etc. Genetic testing is mandatory for the diagnosis of HC. Case Presentation: The aim of this case report is to underline the importance of a multidisciplinary approach for a precise diagnosis. A 17-year-old male teenager with a past medical history of multiple abdominal surgical interventions presented to the emergency department for diarrhea and abdominal pain. The physical examination revealed weight deficit, café-au-lait macules, axillary and inguinal freckling, and mild abdominal tenderness. Laboratory evaluation revealed high CRP, mild leucocytosis and lymphopenia. The surgical consult ruled out an acute surgical cause. The abdominal X-ray, stool culture, fecal cytology, urinalysis and urine culture were negative, but ferritin level and serum iron level were very high. Ophthalmological consult described a Lisch nodule. Multiplex Ligation-dependent Probe Amplification (MLPA) genetic testing found no deletions or duplications within the *NF1* gene. The clinical evolution was favorable under third-generation cephalosporin, but the ferritin and serum iron levels remain elevated. The Whole-Exome Sequencing (WES) identified a heterozygous pathogenic variant in the *NF1* gene, i.e., chr17:29560075CA>C, *NF1*(NM_001042492.3):c.3556del; p.(Ile1186SerfsTer29); rs2151435407, but also revealed a homozygous pathogenic variant in the *HFE* gene, i.e., chr6:26090951C>G, *HFE*(NM_000410.4):c.187C>G; p.(His63Asp), rs1799945, which is associated with hereditary HC. Conclusions: The overlapping dermatological findings of both NF1 and HC might lead to diagnostic delays. The holistic approach to all pediatric patients presenting with particular hyperpigmentation involves thorough laboratory investigations, neurological, ophthalmological, cardiological and genetic examinations. In the absence of DNA Copy Number Variations (CNVs) that can be identified rapidly and cost-effectively by MLPA, sequencing is indicated to analyze small DNA variations, especially when two conditions with similar clinical pictures coexist.

## 1. Introduction

Type I neurofibromatosis (NF1), also known as von Recklinghausen disease, represents an autosomal dominant inherited genodermatosis predisposing to tumor occurrence, caused by mutations in the NF1 gene on chromosome 17q11.2 [1]. The incidence of NF1 is 1:3000 [2] and is clinically characterized by the impairment of skin pigmentation resulting in the appearance of café-au-lait macules, skin-fold freckling and dermal neurofibromas associated with neuro-psychiatric involvement (hyperactivity, learning and attention deficits), Lisch nodules, plexiform neurofibromas, optic pathway gliomas and skeletal abnormalities [1].

The diagnostic criteria for NF1 were initially defined in 1998 by a National Institutes of Health Consensus Conference and the positive diagnosis was defined as the presence of two or more of the following features: at least six café-au-lait macules with the greatest diameter of more than 5 mm before puberty, and over 15 mm in postpubertal individuals; Crowe’s sign—inguinal or axillary freckles; at least two Lisch nodules; at least two neurofibromas regardless of the type or at least one neurofibroma; optic glioma; the presence of a distinctive osseous lesion (thinning of the long bones cortex with or without pseudarthrosis, sphenoid wing dysplasia); and positive family history of NF1 in a first-degree relative [3]. Taking into account that over time several clinical entities were found to have overlapping signs, the diagnostic criteria were revised in 2021 and were divided into two situations. (A) In the absence of a positive NF1 diagnosis in one of the parents, the diagnosis is established according to the Consensus from 1988, preserving the same criteria with two additional criteria: the presence of at least two choroidal anomalies consisting of bright patchy nodules proved by optical coherence tomography or near-infrared reflectance, and the carriage of a heterozygous pathogenic variant in the *NF1* gene with a variant allele frequency of 50% in apparently normal tissue like white blood cells. (B) In terms of a child whose parent meets the previously mentioned diagnostic criteria, the positive diagnosis of NF1 can be established in the presence of one or more of the criteria listed in (A) [4]. The revision of the NF1 diagnostic criteria was introduced to facilitate its diagnosis in young children who present only with skin pigmentation anomalies or a positive family history of NF1 and did not meet the formerly defined diagnostic criteria from 1988.

All patients diagnosed with NF1 should benefit from age-specific annual monitoring and proper education considering the wide spectrum of manifestations and unpredictable degree of severity. Moreover, dermatological and surgical specific treatments along with behavioral and neuropsychiatric therapies should be provided depending on the patient’s symptoms [1].

Hemochromatosis (HC) is a genetic disorder consisting of systemic iron overload due to a defect in hepcidin, which is known to reduce iron transport into plasma, excluding both acquired causes of iron overload (excessive iron supplementation, multiple blood transfusions, or dyserythropoiesis), and other genetic disorders that result in systemic iron overload due to other causes than primary hepcidin deficiency (atransferrinemia, divalent cation transporter 1-related iron overload, ferroportin disease, or aceruloplasminemia) [5]. The mutations that cause HC are most commonly located in the *HFE* gene (homozygous p.Cys282Tyr, C282Y) and affect almost exclusively white individuals, resulting in *HFE*-associated HC, also known as type 1 HC. Nevertheless, well-documented mutations in genes other than *HFE* can also trigger systemic iron overload, leading to non-*HFE* HC that can be diagnosed in both white and non-white individuals. Eventually, both *HFE*- and non-*HFE*-associated HC lead to the accumulation of iron in parenchymal cells, especially hepatocytes, cardiomyocytes and pancreatic cells.

In terms of clinical picture, symptoms are usually lacking below the age of 30–40 years in men and 40–50 years in women, being frequently miscellaneous and non-specific, and subsequently explaining the diagnostic delays [6,7,8]. Nevertheless, when the symptoms occur, chronic fatigue predominates in the clinical picture associated with joint pain (arthritis of the second and third metacarpophalangeal joints and ankles are highly suggestive of HC); spontaneous fractures; dermatological signs such as hyperpigmentation (dark spots, freckling, tanned skin, grayish skin pigmentation), melanoderma, skin dryness and nail impairment; hepatomegaly, cirrhosis, or hepatocellular carcinoma; diabetes, adrenal insufficiency, or hypopituitarism; and cardiac rhythm disorders or heart failure [5]. It is worth mentioning that the presence of anemia along with excessive iron accumulation suggests other genetic disorders such as hereditary aceruloplasminemia, congenital atransferrinemia, or divalent cation transporter 1-related iron overload [9,10,11]. Genetic testing is mandatory for confirming the diagnosis of HC, while liver biopsy is no longer required. Active screening and prevention in all families with a member diagnosed with HC is highly important, as justified by the minimally invasive pattern of the diagnosis and the increased efficiency, simplicity and low-cost treatment, i.e., phlebotomy [5].

Management of HC is based on iron removal therapies such as phlebotomy or chelation therapy (deferoxamine) [12,13], the avoidance of alcohol abuse and oral iron supplements [14], liver cases in rare cases, mostly indicated in the case of hepatocellular carcinoma [15], and hepcidin analogues (more suitable for maintenance therapy) [16,17].

The aim of this case report is to underline the importance of a multidisciplinary approach for a precise diagnosis.

The informed consent was signed by the patient’s mother prior to the publication of this case.

## 2. Case Presentation

### 2.1. Presenting Concerns

We report the case of a 17-year-old male teenager who presented to the emergency department with a 4-day history of diarrhea and abdominal pain, without fever, for which the general practitioner recommended symptomatic treatment.

### 2.2. History

According to the mother, no significant prenatal or perinatal events were recorded; the patient’s birth weight was 3300 g, with no relevant information regarding the family history (hyperpigmentation, tumors, HC or NF1). Affirmatively, the patient’s developmental and psychomotor milestones were according to the age, with no previous neurological and ophthalmological history. The only notable information was that the patient’s school performance was relatively low.

#### Abdominal History and Imaging

The patient’s past medical history was notable for horseshoe kidney and multiple abdominal surgical interventions.

**Figure 1:**
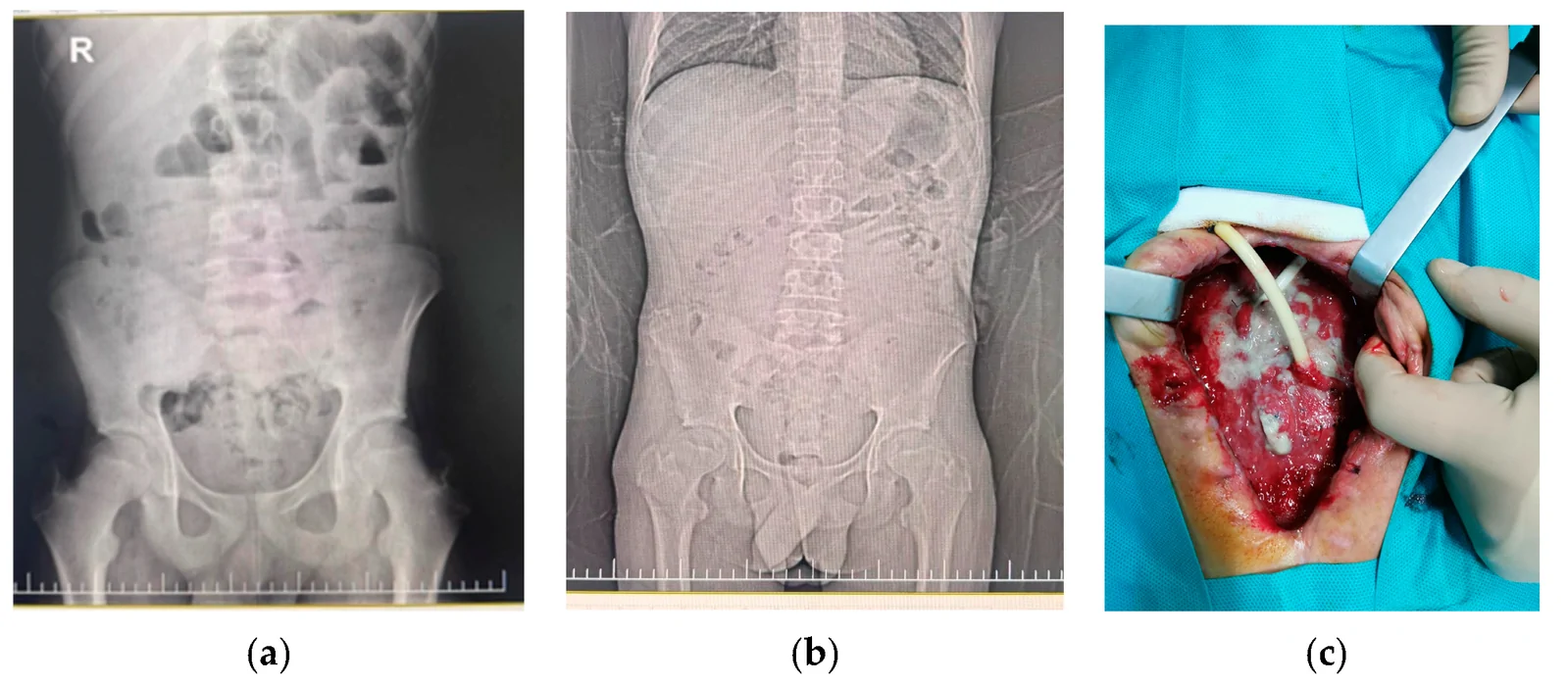
(**a**) *Preoperative* *abdominal X-ray performed for suspected peritonitis and intestinal obstruction prior to surgical intervention*—multiple medium-sized air–fluid levels in the left flank and upper abdominal region; (**b**) *abdominal X-ray 3 months after the 1st intervention:* left abdominal ileostomy, with no air–fluid levels; (**c**) *intraoperative findings*: a granulomatous intestinal mass with multiple perforations and enterocutaneous fistulas.

The patient had multiple abdominal surgical interventions three years before the current admission for generalized peritonitis, appendicular abscess, recurrent bowel occlusions caused by adhesions, mid-abdominal entero-cutaneous fistula and ileal resection with ileostomy (Figure 1). His most recent surgical procedure was performed 5 months prior to the current admission for the closure of the stoma.

Additionally, during his clinical course, NF1 was suspected, but the diagnosis was never confirmed due to the parents’ lack of compliance.

### 2.3. Physical Examination

The clinical examination revealed pale skin with multiple disseminated café-au-lait macules on the trunk (≥6), axillary and inguinal freckling, multiple post-operative hyperpigmented scars on the abdomen, mild abdominal tenderness on palpation, accelerated bowel movements with watery stools and anorexia. We also noted mild intellectual disability. The patient weighed 47 kg, his height was 157 cm, and he had a body mass index (BMI) of 19.1 kg/m^2^, 18th percentile.

### 2.4. Diagnosis and Assessment

The initial laboratory evaluation revealed a marked inflammatory syndrome (C-reactive protein, CRP 2.70 mg/dL, reference range 0–0.5 mg/dL), mild leukocytosis and lymphopenia, with the remaining parameters within normal limits. Given the patient’s medical history and the risk of potential complications, we referred the patient for a surgical consult, which ruled out an acute surgical cause of the gastrointestinal symptoms. The abdominal X-ray showed no significant abnormalities, and the infectious disease consult recommended empirical intravenous antibiotic therapy. In order to establish the etiology, stool samples were collected for viral antigens, stool culture and fecal cytology, all of which were negative. Urinalysis and urine culture were likewise negative.

Taking into account the patient’s history and the laboratory findings, we initiated antibiotic therapy (3rd-generation cephalosporin), with favorable clinical evolution after 48 h, and serial laboratory monitoring demonstrated a decrease in CRP levels; however, a severely elevated ferritin level (1361 ng/mL) and increased serum iron levels (initially 70 µg/dL, subsequently increasing to 150 µg/dL) were detected (Table 1). The clinical course under antibiotic and symptomatic treatment was favorable, with the resolution of gastrointestinal symptoms and normalization of CRP.

In light of the suspected diagnosis of neurofibromatosis, the patient was referred for an ophthalmological examination, which revealed a Lisch nodule at the 5 o’clock position in the right eye. The ear–nose–throat (ENT) examination showed no pathological findings (Table 2). Furthermore, abdominal and pelvic magnetic resonance imaging (MRI) revealed no significant abnormalities.

**Table 2:** Clinical and laboratory comparison of NF1 and HC.

	Type I Neurofibromatosis (NF1)	Hemochromatosis (HC)	Patient
**Clinical signs**			
*Constitutional symptom* [18,19]	-	Chronic fatigue	-
*Skin involvement* [20,21,22,23,24,25,26,27]	Café-au-lait macules	Hyperpigmentation (dark spots, tanned skin, grayish skin pigmentation), melanoderma, alopecia, ichthyosis, skin atrophy	≥6 café-au-lait macules
	Crowe’s sign—inguinal and/or axillary freckles	Freckling	Inguinal and axillary freckles
	Dermal neurofibromas or plexiform neurofibromas	Skin dryness and nail impairment	-
*Neuro-psychiatric involvement* [21,28,29]	Seizures, peripheral neuropathy, Hyperactivity, ADHD, learning and attention deficits	Rare: depression, anxiety, tremors, movement disorders	Mild intellectual disability
*Ophthalmological involvement* [1,30]	Lisch nodules	-	Lisch nodule in the right eye
	Optic gliomas	-	-
*Skeletal involvement* [5,8,19,21,30,31]	Skeletal abnormalities (orbital and tibial dysplasia, scoliosis)	Joint pain, arthritis of the second and third metacarpophalangeal joints and ankles, spontaneous fractures, osteoporosis	-
*Muscular involvement* [18,19]	-	Weakness, decreased muscle mass	-
*Gastrointestinal involvement* [19,21,32]	Gastrointestinal stromal tumors (rare)	Hepatomegaly, cirrhosis, high liver enzymes, chronic hepatic failure	-
*Cardiovascular involvement* [19,21,33]	Arterial hypertension, cardiomyopathy	Arrhythmias (very rare), heart failure, cardiomyopathy, portal hypertension	-
*Endocrine system* [19,21]	-	Hypogonadism, testicular atrophy, amenorrhea, hyperglycemia, diabetes mellitus, hypopituitarism, hypothyroidism	-
*Metabolic alteration* [28]	Lower body mass index (BMI), reduced stature, reduced triglyceride stores, decreased bone mineral density	-	Low BMI
**Laboratory**			
	No specific lab	High ferritin and serum iron level, elevated transferrin saturation	High ferritin and serum iron level
**Genetic tests**			
	Pathogenic variant in the *NF1* gene located on chromosome 17q11.2	Pathogenic variant in the *HFE* gene, C282Y	*NF1* gene, *NF1*:c.3552delA, rs2151435407 and *HFE* gene, *HFE*:c.187C>G, rs1799945

### 2.5. Clinical Course and Genetic Testing

Based on these findings, a genetic consult and Multiplex Ligation-dependent Probe Amplification (MLPA) genetic testing were performed, but no deletions or duplications were found within the *NF1* gene.

Given the persistently elevated ferritin and rising serum iron levels, a brain MRI was performed, with no abnormal images. The laboratory tests repeated 1 month after the 1st admission pointed out the persistence of hyperferritinemia and increased serum iron (ferritin: 1421 ng/mL, serum iron: 209 µg/dL).

Based on all the above, we discussed this with the genetics specialist, and we decided to perform Whole-Exome Sequencing (WES). The WES was performed at BGI Genomics Medical Laboratory (XOME Express, HW1101, BGI Europe, Symfonivej 34, 2730 Herlev, Denmark). The WES was performed with the DNBSEQ platform and cPAS/DNA nanoballs technology and included CNV analysis as described by BGI Laboratory [34]. The quality results are presented below in Table 3.

The pathogenicity was classified by an in-house automatic algorithm according to ACMG guidelines [35]. The results were also evaluated by our geneticist and all relevant VUS were manually reviewed (ClinVar, VarSome and Franklin databases were used).

The WES analysis identified a heterozygous pathogenic variant in the *NF1* gene, chr17:29560075CA>C, *NF1*(NM_001042492.3):c.3556del; p.(Ile1186SerfsTer29); rs2151435407. This frameshift variant, occurring in exon 27 of the gene, was reported to be pathogenic and associated with Neurofibromatosis type 1. Allelic depth for the mentioned variant was 232, and the allelic ratio was 0.52. According to the ClinVar database, the mentioned variant is not present in population databases (gnomAD: no frequency), but this premature translational stop signal has been observed in individual(s) with NF1 [36]. The database ClinVar contains an entry for the mentioned variant (Variation ID: 1048693). In consequence, this *NF1* variant has been classified as pathogenic.

The second pathogenic variant identified was in a homozygous genotype in the *HFE* gene, chr6:26090951C>G, *HFE*(NM_000410.4):c.187C>G; p.(His63Asp), rs1799945. This missense variant, occurring in exon 2 of the gene, was reported to be pathogenic and associated with hereditary HC (noting that this variant generally exhibits variable and lower penetrance). Allelic depth for the mentioned variant was 202, and the allelic ratio was 0.92. This variant may explain both hyperferritinemia and systemic iron overload. The databases mentioned before reported that the variant may be associated with disease; however, the variant appears to have very low penetrance, as the majority of homozygous or compound heterozygous individuals with this variant do not exhibit clinical symptoms of HC, despite some cases having elevated serum ferritin and transferrin saturation levels. This highlights the importance of epigenetics and other factors impacting the manifestation/progression of the disease.

Based on the findings provided by the WES, the family benefited from genetic counseling. Nevertheless, the parents refused the targeted testing for the mentioned variants in order to determine whether those are de novo or germline variants. The family had no history of NF1 or HE, as we mentioned before.

Shortly after the WES testing, the patient reached 18 years of age and was transitioned to the adult specialty care department.

## 3. Discussion

NF1 and HC represent two complex, multi-systemic genetic disorders sharing skin impairment as a major, common clinical trait. Thus, the predominant dermatological manifestation for both NF1 and HC is represented by hyperpigmentation. In terms of NF1, skin impairment is an important diagnostic criterion consisting either of café-au-lait macules or freckling [3,4], while in patients with HC, hyperpigmentation can manifest as dark spots, freckling, hyperpigmented scars, grayish skin pigmentation or tanned skin [20]. Our patient also presented with hyperpigmentation consisting of multiple café-au-lait macules/dark spots, axillary and inguinal freckling, and hyperpigmented abdominal scars. Moreover, according to a recent review involving 148 patients with hereditary or idiopathic HC, the most common signs and symptoms were increased liver size, weight loss, cachexia, anorexia, abdominal pain, malaise or fatigue [20]. Gastrointestinal symptoms are rather nonspecific among HC patients, including abdominal pain, bleeding, obstruction and protein-losing enteropathy, but are not uncommon in those with NF1, being caused by a wide spectrum of gastrointestinal and related intra-abdominal lesions such as neurofibromas, schwannomas, gastrointestinal neurofibromatosis, periampullary neoplasms, gastrointestinal stromal tumors, proliferative mesenchymal precursor lesions, NF1-associated vasculopathies, as well as the coexistence between certain of the previously mentioned tumors [37,38]. Abdominal pain was also present in our patient as a common clinical feature of both conditions, but our patient had a positive history of multiple surgical interventions for recurrent bowel occlusion without an obvious cause at that time, which is definitely more suggestive of NF1. Aside from abdominal pain, anorexia was also present in our case.

Nevertheless, the association between these two conditions is very uncommon and might result in diagnostic delays or it might even mislead the diagnosis. Thus, genetic testing, particularly WES with CNV analysis, should be the sole reliable diagnostic method. In our case, the severely increased ferritin level was initially interpreted as a marker of infection, but its persistence prompted further investigation being unspecific for NF1, which initially seemed the most likely diagnosis based on the patient’s history, clinical and paraclinical findings.

Concerning the association of genotype–phenotype in patients with NF1, it was previously documented that mutations causing the loss of a single nucleotide located in the *NF1* gene, like in our case, may be associated with mild phenotypes of NF1 when compared to NF1 patients carrying large deletions which frequently involve facial dysmorphism, large hands and feet, muscular hypotonia, joint hyperflexibility, significant cognitive delay, cardiovascular malformations, increased frequency and number of subcutaneous, plexiform and spinal neurofibromas, internal neurofibromas, as well as malignant peripheral nerve sheath tumors [39]. Nevertheless, similarly to other genetic syndromes, echocardiography is mandatory in all children with NF1 at the time of diagnosis [40]. In consequence, we can expect a milder phenotype of NF1, but genotype–phenotype associations have not been described until now. However, the geneticist and neurologist will monitor the patient’s clinical evolution and perform the necessary clinical and paraclinical investigations.

The two most common *HFE* variations associated with HC are a G-to-A transition at nucleotide 845 resulting in a change of cysteine to tyrosine at amino acid 282 (C282Y), and a C-to-G transition at nucleotide 187 leading to a substitution of histidine with aspartic acid at amino acid 63 (H63D) [41]. According to the literature, up to 90% of the patients diagnosed with HC are homozygous for the *C282Y* variant, and only 5% of them are compound heterozygous for both variants C282Y and H63D [42,43,44]. Although the C282Y HC variant is more frequently associated with HC, the H63D variant is more common in the general population (8.1% versus 1.9%) and its frequency is higher in European individuals (14.9%) when compared to Asian, African, American or those originating from the Middle East [43,44,45]. Our patient, originating from Romania (Europe), tested positive for *HFE:c.187C>G*, *rs1799945*, i.e., the H63D variant, a variant described to have variable and lower penetrance, which might be a potential explanation for his normal liver function and no imaging signs of iron storage within the brain. An interesting finding reported in the literature is that HC variants, especially the H63D ones, might be risk factors for neurodegenerative diseases due to their association with impaired iron homeostasis, increased oxidative stress, glutamate release, and eventually, aberrant inflammatory responses [46]. As we mentioned before, the majority of patients with this variant in homozygous or compound heterozygous genotype do not develop complete HC symptoms, but some cases were reported to have elevated serum ferritin and/or transferrin saturation levels, defining biochemical HC. This highlights the importance of epigenetics and other factors impacting the manifestation/progression of the disease and the importance of patient monitoring.

To the best of our knowledge, this is the first case ever reported in the literature of a patient carrying two mutations confirming the coexistence of NF1 and HC.

## 4. Conclusions

The overlapping dermatological findings of both NF1 and HC might lead to diagnostic delays if not properly approached, especially in children where the complementary clinical picture is commonly unspecific. Thus, a holistic approach to all pediatric patients presenting with particular hyperpigmentation involving thorough laboratory investigations, as well as neurological, ophthalmological, cardiological and genetic examinations, is definitely the most accurate pathway towards the correct diagnosis. MLPA testing might be used for both NF1 and HC as a first genetic testing method for detecting large deletions/duplications, but if no CNVs are described, sequencing (including WES) is recommended for detecting small variants in children with suggestive clinical features or paraclinical parameters. Moreover, in peculiar cases with contradictory clinical and/or laboratory findings, routine laboratory tests might not be enough for identifying the precise cause of all signs/symptoms, much less in extremely rare cases when two conditions with similar clinical pictures coexist.

## Figures and Tables

**Table 1:** Laboratory data.

Range from Admission	1st Day	3rd Day	7th Day	1 Month	2 Months	Reference Range
CRP	**19.6**	**2.70**	-	-	-	0–0.5 mg/dL
Ferritin	-	**1361**	**-**	**1351**	**1421**	15–120 ng/mL
Serum iron	70	150	150	**180**	**209**	31–168 µg/dL
ESR	-	-	6	6	6	3–10 mm/h
UIBC	-	-	**<25**	-	-	69–240 µg/dL

ESR—Erythrocyte Sedimentation Rate; UIBC—Unsaturated Iron-Binding Capacity. The values highlighted in bold indicate abnormal laboratory findings.

**Table 3:** Quality results of genetic testing.

Raw Data Yield (Mb)	30,676.49
Length (bp)	35,735,556
Coverage	99.80%
Mean depth (×)	446.35
Proportion (Mean Depth > 4×)	99.38%
Proportion (Mean Depth > 10×)	99.19%
Proportion (Mean Depth > 20×)	99.11%
Proportion (Mean Depth > 30×)	99.06%

## Data Availability

The data presented in this case report are available on request from the corresponding author. The data are not publicly available due to patient privacy and ethical restrictions.

## Abbreviations

The following abbreviations are used in this manuscript:

BMI	Body mass index
NF1	Type I neurofibromatosis
HE	Hemochromatosis
CRP	C-reactive protein
ENT	Ear–nose–throat
WES	Whole-Exome Sequencing
